# Effects of larval extracts from identified *Protaetia brevitarsis seulensis* against benign prostatic hyperplasia induced by testosterone in rats

**DOI:** 10.1002/fsn3.2460

**Published:** 2021-08-06

**Authors:** Yun‐Soo Seo, Na‐Rea Shin, Hyeon Hwa Nam, Jun‐Ho Song, Byeong Cheol Moon, Goya Choi, In‐Sik Shin, Joong‐Sun Kim

**Affiliations:** ^1^ Herbal Medicine Resources Research Center Korea Institute of Oriental Medicine Naju‐si Korea; ^2^ College of Veterinary Medicine and BK21 Plus Project Team Chonnam National University Gwangju Korea

**Keywords:** benign prostatic hyperplasia, dihydrotestosterone, *Protaetia brevitarsis seulensison*, testosterone

## Abstract

*Protaetia brevitarsis seulensis* is an animal‐based medicine used traditionally in China, Japan, and Korea to treat hepatic disorders; it has been shown to possess various pharmacological effects such as antibacterial and antioxidant activities. In this study, we investigated the effects of *P*. *brevitarsis* on a testosterone‐induced benign prostatic hyperplasia (BPH) rat model. To establish the BPH model, the animals were administered a subcutaneous injection of testosterone daily for 28 days. *P*. *brevitarsis* was administered by oral gavage at doses of 12.5, 25, and 50 mg/kg for 28 days, along with testosterone injection. *P*. *brevitarsis* treatment markedly decreased the absolute and relative prostate weight of BPH animals. The levels of dihydrotestosterone was reduced in *P*. *brevitarsis*‐treated animals compared to those in the BPH animals. Histological analysis of the prostate showed that *P*. *brevitarsis* treatment effectively suppressed the testosterone‐induced hyperplasia of prostatic epithelial cells, which was accompanied by reductions in the PCNA and Ki‐67 expressions in prostatic tissues. These results indicate that *P*. *brevitarsis* effectively suppresses testosterone‐induced development of BPH, and thus, is a potential therapeutic agent for BPH.

## Graphical abstract

Treatment with Protaetia brevitarsis seulensis extracts (PBE) reduced prostate weight, dihydrotestosterone levels of benign prostatic hyperplasia (BPH) animals. Furthermore, PBE attenuated the testosterone‐induced hyperplasia and proliferation of prostatic epithelial cells. These results indicate that *P*. *brevitarsis* is a potential therapeutic agent for BPH.

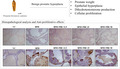

## 1 INTRODUCTION

Benign prostatic hyperplasia (BPH) is a nonmalignant proliferative disorder of the prostate. The development of BPH usually occurs after 40 years of age and its prevalence gradually increases with increasing age (Shin et al., 2012a). Its prevalence is nearly 50% in men over the age of 50 and reaches 80% in men over the age of 90 (Lim et al., 2018). BPH is featured as a hyperplasia of the stromal and epithelial cells of the prostate, thereby increased prostate size and caused lower urinary tract symptoms such as dysuresia (Jeon et al., 2017). Patients with BPH suffer mentally and physically because of increased urgency and frequency of urination caused by the constriction of the urethra due to pressure from the enlarged prostate (Ammar et al., 2015).

Benign prostatic hyperplasia is induced by various factors, including androgens, inflammation, and reactive oxygen species (Naber & Weidner, 2000). Of these factors, androgens are considered important in mediating the development of BPH (Huhtaniemi & Forti, 2011). Androgens such as testosterone and dihydrotestosterone (DHT) induce prostatic enlargement via binding to the prostate androgen receptor; specifically, prostatic 5α‐reductase converts testosterone to DHT, which has a higher binding capacity to the androgen receptor than testosterone. As DHT binds to the androgen receptor, it is translocated into the nucleus and induces the expression of the gene associated with prostate growth and differentiation (Ub Wijerathne et al., 2017). Based on these pieces of evidence, two classes of medications to treat BPH have been developed 5α‐reductase inhibitors and α1‐adrenergic receptor antagonists. Dutasteride, as a 5α‐Reductase inhibitor inhibits the conversion of testosterone to DHT by suppressing 5α‐reductase, whereas tamsolusin, as an α1‐adrenergic receptor antagonist improves the clinical signs of BPH by relaxing the prostate smooth muscle and the neck of the bladder (Shin, Lee, Ha, et al., 2012). The use of these two classes of medications is limited because of their adverse effects, including decreased libido, ejaculatory dysfunction and upper respiratory tract infection (Bullock & Andriole, 2006). Therefore, alternative medicines for the treatment of BPH are being developed continuously (Akanni et al., 2017; Ammar et al., 2015; Jena et al., 2016; Kim et al., 2015).

The white‐spotted flower chafer (Protaetia brevitarsis seulensis) is a traditional animal‐derived medicine used in China, Japan, and Korea to treat hepatic disorders, dysuresia, breast cancer, and various inflammatory diseases (Lee, Hwang, et al., 2017; Yeo et al., 2013). *P. brevitarsis* belongs to the family Scarabaeidae of the order Coleoptera and is widely distributed in East Asia (China, Japan, Korea, and Taiwan) and Europe (Yeo et al., 2013). It has various pharmacological effects, including antioxidant, antithrombotic, and anticancer activities (Lee, Lee, et al., 2017; Suh & Kang, 2012; Yoo et al., 2007). However, until now, no study has explored the effect of *P. brevitarsis* on the development of BPH. Therefore, we investigated the effect of *P. brevitarsis* on the testosterone propionate‐induced BPH rat model. To better understand the pharmacological properties of *P. brevitarsis*, we evaluated the DHT levels in the serum and the expression of a cell proliferation‐related protein in the prostate.

## 2 MATERIALS AND METHODS

### 2.1 Preparation of *P*. *brevitarsis*

The larval extracts from *P. brevitarsis* were obtained from Kwangmyongdang Co. and samples (manufacture's No. K2281201707) were deposited in the Korean Herbarium of Standard Herbal Resources (Index Herbariorum code KIOM) at the Korea Institute of Oriental Medicine, Naju, Korea (medicinal ID: 2–18–0111) authenticated based on the macroscopic characteristics described by Dr. Goya Choi of Korea Institute of Oriental Medicine.

Moreover, to discriminate the species of herbal medicine, Holotrichia was used in this study. We amplified and sequenced COI DNA barcode regions, an universal DNA barcode used for identifying animal species, from randomly selected five larvae and compared the sequence identity with other COI sequences registered in the GenBank using BLAST (Basic Local Alignment Search Tool) analysis (Che et al., 2012; Lee et al., 2021). To confirm the sequence identity and species of our samples, furthermore, comparative sequence analysis was carried out between the COI sequences of five samples and other closely related insect species registered in the GenBank including Protaetia brevitarsis (KC775706), P. affinis (KM286290), P. speciosissima (KM286124 and KJ908751), P. lunubris (KM286218, KU908751, and KU916955), P. marmorata (KJ964464), P. cuprea (DQ295301), P. aurichalcea (KM033437), and P. morio (KY827323).

The dried crude material (887.4 g) was extracted twice with 15 L water (with a 2 hr reflux), and the extract (242.8 g) was filtered using cellulose Chromatography papers (Whatman^®^ 3 MM papers, Sigma‐Aldrich). And the materials was lyophilized at −70℃, and stored at 4℃. The yield of the dried extract was approximately 27.4% (w/w).

### 2.2 Experimental animals

Male Sprague Dawley rats (specific‐pathogen‐free, 8 weeks old, 200–220 g) were obtained from Samtako Co. and were used after 2 weeks of quarantine treatment and acclimatization. The animals were housed in a controlled environment (temperature 18–23℃, humidity 40%–60%, standard laboratory diet from Samyang Feed and water ad libitum). All experimental procedures were approved by the institutional Animal Care and Use Committee of Chonnam National University (CNU IACUC‐YB‐R‐2016–38).

### 2.3 Testosterone‐induced BPH rat model and drug treatment

The BPH animal model was constructed by previous report (Jeon et al., 2017). To induce BPH, testosterone propionate (TP, 3 mg/kg, Tokyo Chemical Ins. Co.) dissolved in corn oil (Sigma‐Aldrich) was administered to animals by subcutaneous injection. The *P. brevitarsis* extract (PBE; 12.5, 25, and 50 mg/kg) and 10 mg/kg finasteride (Sigma‐Aldrich; included as a positive control) were administered to animals by oral gavage 1 hr after TP injection for 4 weeks. The animals were divided into the following groups with five rats each: VC, animals receiving corn oil and PBS; BPH, animals receiving TP and PBS; Fin, animals receiving TP and finasteride; BPH + PBE 12.5, 25, and 50, animals receiving TP and PBE at 12.5, 25, and 50 mg/kg, respectively; and PBE, animals receiving only PBE (50 mg/kg). The PBE group is designed to evaluate the hepatic toxic effects of PBE via serum biochemistry. Twenty‐four hr after the last treatment, the animals were sacrificed and blood samples were obtained from the caudal vena cava. Whole prostates were immediately sampled and weighted. Relative prostate weight was expressed as the proportion of organ weight to body weight.

### 2.4 Measurement of the levels of dihydrotestosterone in serum

Serum was separated from whole blood via centrifugation (200 *g*, 10 min). The quantitative analysis of DHT in the serum were determined using a commercial enzyme‐linked‐immunosorbent assay (ELISA) kit (ALPCO) according to the manufacturer's protocols. The absorbance was measured at 450 nm using spectrophotometer (Bio‐Rad Laboratories). The value were expressed as per ml in serum.

### 2.5 Immunoblotting

Prostate tissue was homogenized with a tissue lysis/extraction reagent (Sigma‐Aldrich) and protein concentration was determined using Bradford reagent (Bio‐Rad Laboratories). Immunoblotting was performed according to methods described previously (Ko et al., 2018). Equal amounts of protein (30 μg) were heated at 100℃ for 5 min, loaded onto 10% SDS‐PAGE gels, and electrophoresed. The following primary antibodies and dilutions were used: anti‐PCNA (1:1,000 dilution, Abcam) and anti‐β‐actin (1:2000 dilution, Cell Signaling). To evaluate the proportion of protein expression, densitometric band values were determined using a Chemi‐Doc imaging system (Bio‐Rad Laboratories).

### 2.6 Histological analysis of prostate

The prostate tissue was embedded in paraffin and sectioned to a thickness of 4 μm. The sections were stained with hematoxylin and eosin (BBC Biochemical) to determine histological alteration of the prostate. Histological analysis was performed using a light microscope (Leica, Wentzler) manually and in a completely blinded manner.

### 2.7 Immunohistochemistry of prostate

To determine the Ki‐67 expression (Santa Cruz Biotechnology) on the prostate, we conducted an immunohistochemical analysis using a commercial kit (Vector Laboratories). The procedure of ICH was performed as previously described (Ko et al., 2018). Each sample was evaluated under a light microscope (Leica) in a completely blinded manner.

### 2.8 Serum biochemical analysis

To investigate the toxic effect of PBE, aspartate aminotransferase (AST) and alanine aminotransferase (ALT) activities were calculated using the Fuji Dri‐Chem 4000i automatic analyzer (Fujifilm Co.).

### 2.9 Statistical analysis

Data are shown as means ± standard deviations. Significance was evaluated using analysis of variance, and when tests showed a significant difference among groups, the data were analyzed by Dunnett's Method. The significance was set at *p* < .05 and *p* < .01.

## 3 RESULTS

### 3.1 The effects of PBE on body weight change

As shown in Table 1, bodyweight did not differ significantly among the experimental groups. Only the PBE‐treated group (PBE) did not differ significantly from the vehicle control group.

**TABLE 1:** Body weight change

Group	Body weight change (g)				
	Day 1	Day 8	Day 15	Day 22	Day 29
VC	326 ± 10.3	348 ± 12.9	375 ± 15.3	400 ± 19.1	414 ± 20.7
BPH	321 ± 14.7	346 ± 19.5	359 ± 19.5	373 ± 20.8	382 ± 21.9
Fin	311 ± 19.1	338 ± 17.9	353 ± 16.0	371 ± 15.2	380 ± 16.1
BPH + PBE 12.5	335 ± 10.7	365 ± 14.2	379 ± 15.8	392 ± 17.2	405 ± 18.0
BPH + PBE 25	325 ± 11.5	348 ± 13.3	365 ± 13.1	382 ± 9.6	395 ± 11.4
BPH + PBE 50	339 ± 10.5	370 ± 11.2	385 ± 13.5	405 ± 16.5	418 ± 19.9
PBE 50	327 ± 19.7	329 ± 47.9	368 ± 24.2	398 ± 24.9	417 ± 28.9

Note

VC, animals receiving corn oil and PBS; BPH, animals receiving TP and PBS; Fin, animals receiving TP and finasteride, BPH + PBE 12.5, 25 and 50, animals receiving TP and PBE (12.5, 25 and 50 mg/kg, respectively); PBE, animals receiving only PBE (50 mg/kg).

### 3.2 The effects of PBE on absolute and relative prostate weight

**FIGURE 1:**
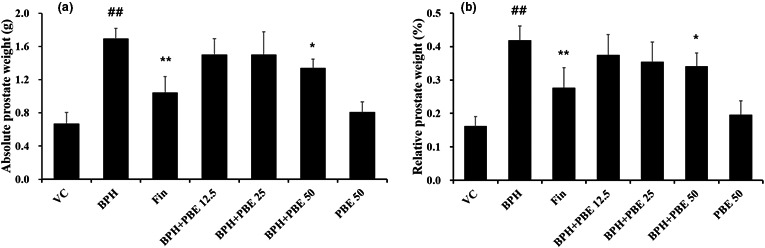
Effects of PBE on absolute and relative prostate weights. At necropsy, whole prostates were immediately removed and weighted. Relative prostate weight was calculated as the ratio of prostate weight to body weight. (a) Absolute prostate weight, (b) Relative prostate weight. VC, animals receiving corn oil and PBS; BPH, animals received TP and PBS; Fin, animals receiving TP and finasteride; BPH + PBE 12.5, 25, and 50, animals receiving TP and PBE at rates of 12.5, 25, and 50 mg/kg, respectively; PBE, animals receiving only PBE (50 mg/kg). Data are presented as mean ± standard deviation (*SD*) (*n* = 6). ##Significant difference at *p* < .01 compared with VC. *,**Significant difference at *p* < .05 and *p* < .01, compared with BPH, respectively

Absolute prostate weight of the BPH group was elevated in comparison to that of the VC group (Figure 1a). But, the absolute prostate weight of finasteride‐treated group was declined in comparison to that of the BPH group. In addition, the absolute prostate weight of BPH + PBE groups was reduced in comparison to that of the BPH group. In particular, the absolute prostate weight of BPH + PBE high‐dose group was significantly declined in comparison to that of the BPH group. However, the relative prostate weight of the BPH group was elevated in comparison to that of the VC group, whereas the relative prostate weight of the BPH + PBE group (high dose) was decreased in comparison to that of the BPH group (Figure 1b). Only the relative prostate weight of the PBE‐treated group did not differ from that of the VC group.

### 3.3 The effects of PBE on serum DHT levels

**FIGURE 2:**
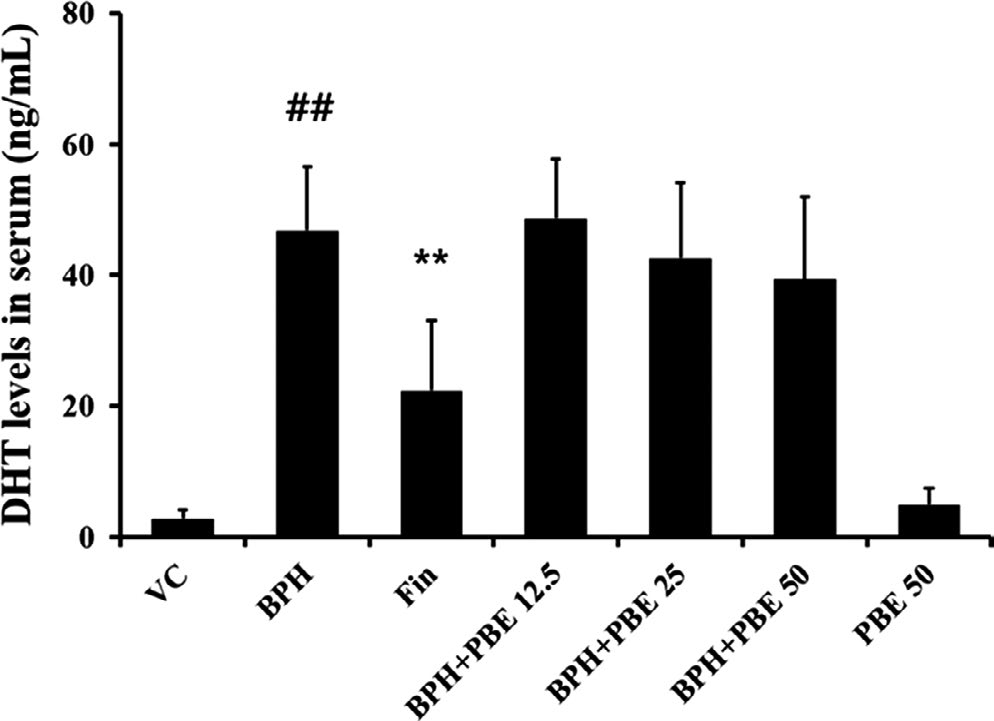
Effects of PBE on the serum DHT level. The levels of DHT in the serum were evaluated using an enzyme‐linked immunosorbent assay (ELISA) kit. VC, animals receiving corn oil and PBS; BPH, animals receiving TP and PBS; Fin, animals receiving TP and finasteride; BPH + PBE 12.5, 25, and 50, animals receiving TP and PBE at rates of 12.5, 25, and 50 mg/kg, respectively; PBE, animals receiving only PBE (50 mg/kg). Data are presented as mean ± standard deviation (*SD*) (*n* = 6). ##Significant difference at *p* < .01 compared with VC. *,**Significant difference at *p* < .05 and *p* < .01 compared with BPH, respectively

The serum DHT level of BPH group was markedly elevated in comparison to that of VC group (Figure 2). In contrast, the serum DHT level of the finasteride‐treated group was significantly declined in comparison to that of the BPH group. The serum DHT levels of the 25 and 50 mg/kg BPH + PBE groups were decreased in comparison to those of the BPH group; however, the difference was not significant. Only the serum DHT levels of the PBE‐treated group differed significantly from those of the VC group.

### 3.4 The effects of PBE on epithelial hyperplasia in the prostate

**FIGURE 3:**
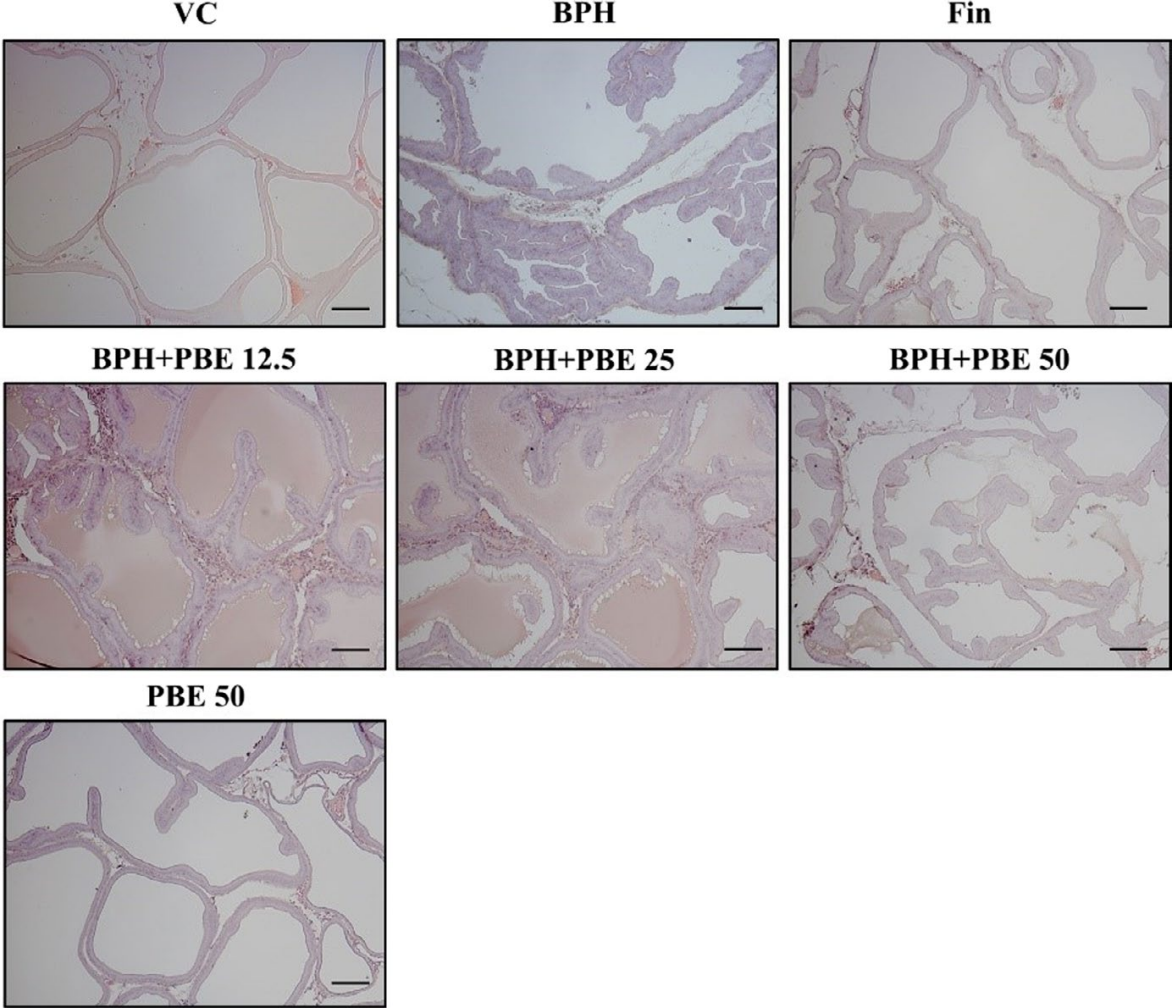
Effects of PBE on epithelial hyperplasia in the prostate. The prostate tissues were embedded in paraffin, sectioned to 4 μm thickness and stained with hematoxylin and eosin (magnification ×200). VC, animals receiving corn oil and PBS; BPH, animals receiving TP and PBS; Fin, animals receiving TP and finasteride; BPH + PBE 12.5, 25, and 50, animals receiving TP and PBE at rates of 12.5, 25, and 50 mg/kg, respectively; PBE, animals receiving only PBE (50 mg/kg)

Prostate samples from the BPH group exhibited extensive epithelial hyperplasia in contrast to the VC group (Figure 3). However, the finasteride‐treated group had a markedly lower level of epithelial hyperplasia compared to that of the BPH group. In addition, epithelial hyperplasia in the 50 mg/kg group of the BPH + PBE treatment was lower than that of the BPH group. The histological structure of prostates from only the PBE‐treated group was similar to that of the VC group.

### 3.5 The effects of PBE on the PCNA expression

**FIGURE 4:**
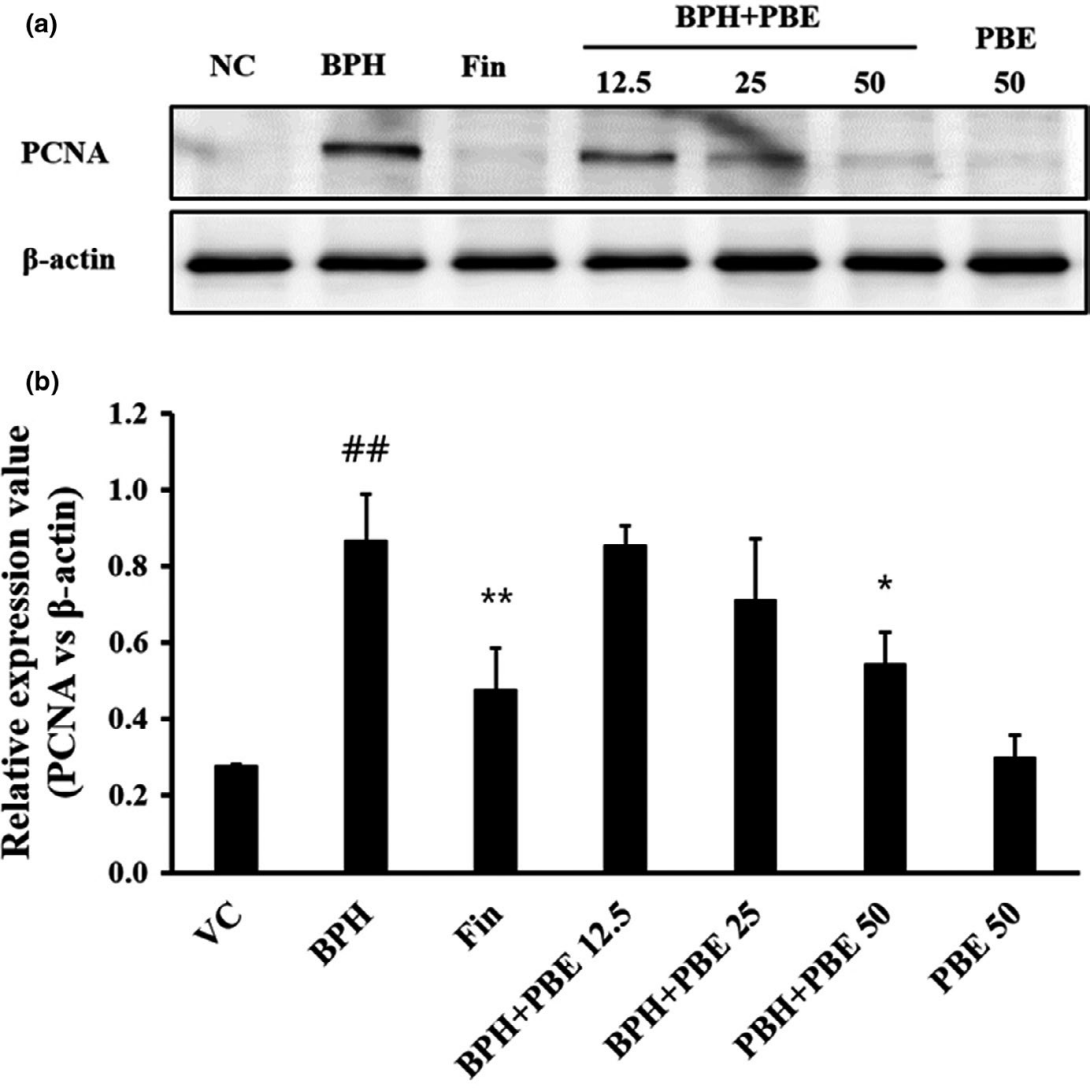
Effects of PBE on the expression of PCNA protein. Immunoblotting was performed according to a previous study (17). The following primary antibodies and dilutions were used: anti‐PCNA (1:1,000 dilution, Abcam) and anti‐β‐actin (1:2000 dilution, Cell Signaling). To evaluate the relative ratio of protein expression, densitometric band values were determined by Chemi‐Doc (Bio‐Rad Laboratories). (a) PCNA expression, (b) relative expression value. VC, animals receiving corn oil and PBS; BPH, animals receiving TP and PBS; Fin, animals receiving TP and finasteride; BPH + PBE 12.5, 25, and 50, animals receiving TP and PBE at rates of 12.5, 25, and 50 mg/kg, respectively; PBE, animals receiving only PBE (50 mg/kg). Data are presented as mean ± standard deviation (*SD*) (*n* = 6). ##Significant difference at *p* < .01 compared with VC. *,**Significant difference at *p* < .05 and *p* < .01 compared with BPH, respectively

The PCNA expression on prostate tissue of the BPH group was markedly increased in comparison to that of the VC group (Figure 4). However, PCNA expression in the finasteride‐treated group declined in comparison to that of the BPH group. Moreover, PCNA levels in the BPH + PBE group were reduced in comparison to those of the BPH group. In particular, PCNA expression in the high‐dose BPH + PBE group was significantly declined in comparison to that in the BPH group. PCNA expression of the only‐PBE‐treated group was similar to that of the VC group.

### 3.6 The effects of PBE on the Ki‐67 expression on the prostates

**FIGURE 5:**
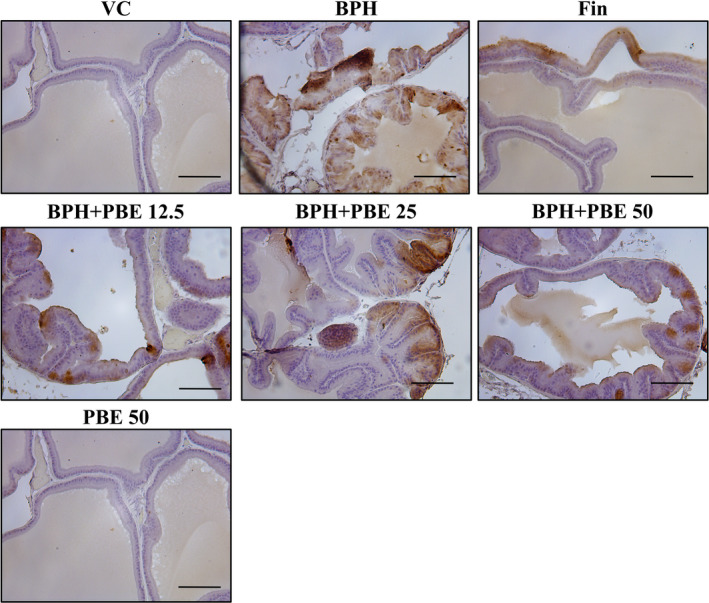
Effect of PBE on the expression of Ki‐67 protein in the prostate. The expression of Ki‐67 in the prostate was evaluated by immunohistochemistry using a commercial kit. The anti‐mouse Ki‐67 antibody was purchased from Santa Cruz Biotechnology and used at a 1:200 dilution. VC, animals receiving corn oil and PBS; BPH, animals receiving TP and PBS; Fin, animals receiving TP and finasteride; BPH + PBE 12.5, 25, and 50, animals receiving TP and PBE at rates of 12.5, 25, and 50 mg/kg, respectively; PBE, animals receiving only PBE (50 mg/kg)

Ki‐67 expression on the prostate of BPH group was higher than that of the VC group (Figure 5). However, the Ki‐67 expression of the finasteride‐treated group was lower than that of the BPH group. Ki‐67 expression of the BPH + PBE group was declined in comparison to that of the BPH group, which was most clearly observable in the high‐dose group. Ki‐67 expression in the only‐PBE‐treated group was similar to that of the VC group.

### 3.7 The effects of PBE on hepatic enzyme activities

**FIGURE 6:**
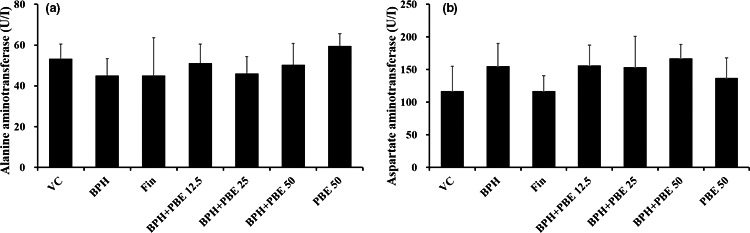
Effect of PBE on hepatic enzyme activities. To investigate the toxic effect of PBE, aspartate aminotransferase (AST) and alanine aminotransferase (ALT) activities were determined using a Fuji Dri‐Chem 4000i automatic analyzer. (a) ALT, (b) AST. VC, animals receiving corn oil and PBS; BPH, animals receiving TP and PBS; Fin, animals receiving TP and finasteride; BPH + PBE 12.5, 25, and 50, animals receiving TP and PBE at rates of 12.5, 25, and 50 mg/kg, respectively; PBE, animals receiving only PBE (50 mg/kg). Data are presented as mean ± standard deviation (*SD*) (*n* = 6)

There were no observable differences in the activities of ALT and AST among the experimental groups (Figure 6a, b, respectively). In addition, the hepatic enzyme activities in the only‐PBE‐treated group did not differ from those in the VC group.

## 4 DISCUSSION

In this study, we investigated the therapeutic effects of PBE on a testosterone‐induced BPH rat model. PBE treatment effectively inhibited prostate enlargement in BPH animals; this was accompanied by a reduction in epithelial hyperplasia of the prostate. In addition, PBE treatment resulted in significantly reduced levels of serum DHT compared to that of BPH animals; this was accompanied by declines in the PCNA and Ki‐67 expressions on the prostate.

The increased weight and size of the prostate, an important feature in the development of BPH, is caused by hyperplasia of stromal and epithelial cell. An enlarged prostate may press against the ureter and ultimately cause dysuresia (Adaramoye et al., 2019). Therefore, prostate weight is a crucial indicator for the diagnosis of BPH and for evaluating the therapeutic effect of a test material (Cai et al., 2018; Rho et al., 2019). In this study, PBE treatment significantly inhibited the testosterone‐induced enlargement of the prostate, indicating that BPH ameliorates the development of BPH. This result is supported by histological analysis of prostate, which is based on epithelial hyperplasia of the prostate being an important feature in BPH. In this study, a marked level of epithelial hyperplasia of the prostate characterized the BPH group, which contrasted much with the prostate of the VC group. However, epithelial hyperplasia of the prostate was inhibited in the BPH + PBE groups, in contrast to the levels exhibited in the BPH group.

The administration of PBE led to the marked decline in the PCNA and Ki‐67 expressions on the prostate. PCNA and Ki‐67 are well‐known as important indicators of cellular proliferation (Ko et al., 2018) and their expression levels are elevated in the prostate of BPH animals (Romar et al., 2016). The levels of these proteins are correlated with the proliferative function of various cells, which makes them valuable markers in the diagnosis of BPH and for evaluating the efficacy of test materials against BPH (Rho et al., 2019; Zhong et al., 2008). Therefore, the reduced PCNA and Ki‐67 expressions on the prostate is related to the inhibition of epithelial hyperplasia of the prostate in the BPH condition. The reduction in PCNA and Ki‐67 expression induced by PBE treatment is an important indicator of the protective effect of PBE against the development of BPH.

Androgens such as testosterone and DHT are crucial mediators in the growth of stromal and epithelial cell of the prostate during BPH progression (Rho et al., 2019). They bind to androgen receptors and activate the transcription of growth factors, resulting in the growth of stromal and epithelial cells of the prostate (Ko et al., 2018). Particularly, DHT, a metabolite of testosterone, is converted by 5α‐reductase and has 10 times more affinity for androgen receptors than testosterone. Therefore, the suppression of DHT production is considered as a crucial strategy for treating BPH (Carson & Rittmaster, 2003). Indeed, finasteride, a recommended medication for BPH, inhibits DHT production by suppressing 5α‐reductase (Youn et al., 2018). Many researchers who have investigated materials to effectively treat BPH have focused on those that inhibit DHT production (Adaramoye et al., 2019; Cai et al., 2018; Rho et al., 2019). Similar to previous findings (Youn et al., 2018), we found that finasteride significantly inhibits the levels of DHT in BPH animals. The administration of PBE also decreased the DHT levels in the serum of BPH animals. These results indicate that PBE inhibits the development of BPH in rats, which is closely associated with the reduced DHT levels in BPH animals.

Herbal and animal product‐based alternative medicines have been traditionally used to treat various disease. However, toxicity and quality control issues associated with alternative medicines for public consumption are areas of increasing concern due to the lack of scientific documentation regarding their toxicity (Shin, Lee, Kim, et al., 2012). In a 13‐week oral repeated dose toxicity study, (Noh et al., 2018) reported that PBE shows no toxicity up to 3,000 mg/kg. In this study, we did not observe any significant changes in bodyweight and hepatic enzyme activities among experimental groups during the experimental period. Therefore, we consider PBE to be a safe material for the treatment of BPH. Although the PBE was effective in treating BPH, the therapeutic effect of PBE was less than finasteride, a commercial drug. Therefore, it is preferable to use PBE as an adjuvant rather than as a therapeutic agent for BPH.

The development of BPH is affected by various factors. Of various factors, ROS is considered to be an important factor (Rho et al., 2019). Overproduction of ROS induces the reductions in antioxidant defense systems resulting in protein, lipid, and DNA damage, which eventually causes the loss of normal cell functions via alteration of intracellular signaling pathway (Romar et al., 2016; Zhong et al., 2008). ROS induces lipid peroxidation and inflammatory responses in prostate leading to the development of BPH (Carson & Rittmaster, 2003; Rho et al., 2019). Based on this evidence, many researchers have investigated therapeutic materials that possess antioxidant properties for treating BPH (Ammar et al., 2015; Jena et al., 2016; Youn et al., 2018).

In our previous study, it has been reported that PBE contains several bioactive compounds including hypoxanthine, uridine, adenine, adenosine, inosine, and benzoic acid (Lee et al., 2021). These compounds known to have biological activities including antiproliferative and antitumor effects (Dinesh et al., 2012). We further investigated that inosine and benzoic acid have significant antioxidative activity in a dose‐dependent manner using our screening system (Lee et al., 2021). There results indicated that PBE, as antioxidant, may be a therapeutic material for treating BPH.

## 5 CONCLUSION

In conclusion, the administration of PBE effectively inhibited several indicators of BPH in animals. This pharmacological effect of PBE may be associated with the suppression of DHT production. We suggest that PBE may be a useful supplement for treating BPH.

## CONFLICT OF INTEREST

The authors declare that they do not have any conflict of interest.

## AUTHOR CONTRIBUTIONS

**Yun‐Soo Seo:** Formal analysis (lead); Investigation (equal); Writing‐original draft (equal); Writing‐review & editing (equal). **Shin Na‐Rae :** Data curation (equal); Formal analysis (equal); Investigation (lead). **Hyeon Hwa Nam:** Formal analysis (equal); Investigation (equal). **Jun‐Ho Song:** Data curation (equal); Formal analysis (equal). **Byeong Cheol Moon:** Funding acquisition (lead); Resources (equal). **Choi Goya:** Formal analysis (equal); Resources (lead). **In‐Sik Shin:** Data curation (equal); Formal analysis (equal); Investigation (equal); Methodology (lead); Writing‐original draft (lead); Writing‐review & editing (equal). **Joong Sun Kim:** Conceptualization (lead); Data curation (equal); Methodology (equal).

## ETHICAL APPROVAL

All experimental procedures were approved by the institutional Animal Care and Use Committee of Chonnam National University (CNU IACUC‐YB‐R‐2016–38) and the animals were cared for in accordance with the NIH Guide for the Care and Use of Laboratory Animals.

## INFORMED CONSENT

Written informed consent was obtained from all study participants.
